# New approaches for the neuroimaging of gene expression

**DOI:** 10.3389/fnint.2015.00005

**Published:** 2015-02-04

**Authors:** Assaf A. Gilad, Galit Pelled

**Affiliations:** ^1^The Russell H. Morgan Department of Radiology and Radiological Science, Johns Hopkins University School of MedicineBaltimore, MD, USA; ^2^Cellular Imaging Section and Vascular Biology Program, Institute for Cell Engineering, The Johns Hopkins University School of MedicineBaltimore, MD, USA; ^3^F. M. Kirby Research Center for Functional Brain Imaging, Kennedy Krieger InstituteBaltimore, MD, USA

**Keywords:** imaging, brain, c-fos, reporter genes, plasticity

With more than 20,000 genes in the human genome now identified and a similar number of genes in the rat and mouse genome known (Gibbs et al., 2004), elucidating their function has become a major challenge. An important contribution to understanding genetic pathways was made by the development of reporter genes. A reporter gene is a gene whose product can be readily detected and either fused to the gene of interest or cloned instead of that particular gene. Optical reporter genes are the most commonly used and widely developed. Throughout the years, multiple genes have been cloned from a variety of organisms that emit light via bioluminescence or fluorescence, in multiple distinguishable wavelengths. An emerging new class of reporter genes encode for proteins with an affinity for radioisotopes or positron emitter probes. These receptors, transporters, or enzymes can provide quantitative images upon administration of suitable radiolabeled probes (Serganova et al., 2007). Magnetic resonance imaging (MRI) reporter genes are unique among all the reporter genes used with the various imaging modalities, since they can provide information about gene expression that can be co-registered with soft tissue anatomical and functional information (Gilad et al., 2007b). A key feature of reporters for MRI (and other non-invasive imaging techniques) is that they enable serial temporal imaging within the same subject. This is particularly useful for studying dynamic processes, for example, the migration of stem cells and progenitors, neuronal plasticity, the mechanisms of development and adaptation, disease progression, and response to trauma or illness, all of which require serial imaging of the same individual over time.

The process of uncovering reporter genes for molecular-genetic imaging usually begins by searching for a gene with desirable imaging properties, such as the green fluorescent protein (GFP) (Shimomura et al., 1962). Advances in recombinant DNA technology have made it possible to clone and express these reporters in both prokaryotic and eukaryotic cells (de Wet et al., 1985; Chalfie et al., 1994). For most candidate reporter genes, a significant improvement in detection efficiency has been achieved after mutations were systematically introduced (Cormack et al., 1996), or by mutations that created variants that could be detected with multiple excitation frequencies (Shaner et al., 2004; Zhao et al., 2005). Directed evolution is a process of generating a library of genes with random mutations and screening for mutants with a superior signal (e.g., enhanced signal), and then repeating the same process using the improved protein as a starting point for mutagenesis until a significant improvement in detection efficiency is achieved. For example, this process was recently applied to the bacterial cytochrome P450-BM3, a putative MRI sensor for dopamine, and improved its sensitivity 100-fold (Shapiro et al., 2010).

Over the past decade, viral vectors have been widely used for gene delivery to the central nervous system (CNS). Viruses of different types (e.g., adeno-associated virus, herpes simplex virus, and lentivirus) have several advantages: they can carry a large amount of genetic material, from 8 to 150 Kb; they can infect a wide range of cells, including non-dividing cells (as most of the cells in the CNS are); and they lead to transient or stable gene expression, depending on the virus type (Davidson and Breakefield, 2003). Lentiviruses are derived from the immunodeficiency virus type 1 (HIV-1). These are retroviruses in which the genetic material is stored as RNA, and the viral particles are coated with a lipid envelope with embedded glycoproteins. The particle size is approximately 100 nm in diameter and can carry 7–8 Kb of genetic material. Lentiviruses induce a minimal inflammatory response and achieve long-term gene expression in the brain (Davidson and Breakefield, 2003; Jakobsson and Lundberg, 2006).

Lentiviral vectors have been used in the CNS to generate models for diseases and for therapy. They are favored because of their ability to infect a vast range of cells and deliver multiple genes simultaneously with long-term expression. Lentivirus has been used to deliver mutant α-Synuclein into the substantia nigra of rats to generate a model of Parkinson's disease (PD) (Lo Bianco et al., 2002). The large transgene capacity of the lentivirus was used to deliver three genes to the biosynthesis pathway of dopamine, which led to physical improvement in a chemically induced rat model of PD (Azzouz et al., 2002). In addition, stable expression of light-activated channels in the mouse's hippocampus was achieved using lentivirus to study neuronal circuits (Zhang et al., 2007). In the majority of these studies, fluorescent genes were used as reporter genes, which usually allow only *ex vivo* analysis or an extremely invasive imaging procedure.

One of the genes in which the ability to monitor and visualize its activity via reporter genes has been highly desired, is *c-fos*, a member of the immediate early gene family (IEG). Initially discovered as a proto-oncogene (Sonnenberg et al., 1989; Sheng and Greenberg, 1990), *c-fos* is well known for being activated in neurons. It is a transcription factor that is transcribed immediately (within minutes) after extracellular activation, which leads to *de novo* synthesis of the *c-fos* protein. The *c-fos* is then dimerized with *c-Jun* (another IEG) and activates the transcription of late-response genes, which are the target genes that result in neuronal plasticity (Sheng and Greenberg, 1990). In the CNS, *c-fos* is induced by several neurotransmitters, Ca2+ influx (Sheng and Greenberg, 1990), and growth factors (NGF, PDGF, and EGF) (Smeyne et al., 1993). The half-life of the *c-fos* mRNA in nature is 15–20 min (Sheng and Greenberg, 1990) and the protein levels peak after two-to-three hours and almost completely decay within 8 h (Sonnenberg et al., 1989). However, once the *c-fos* promoter drives the expression of the reporter gene, the half-life will be dependent on the reporter's mRNA and protein properties. For example, the *in vitro* expression in the CHO (Chinese Hamster Ovary) cell line of mutated green florescent protein (GFP) had a much shorter half-life than the wild type GFP, both of which were under the regulation of the *c-fos* promoter (Bi et al., 2002). It is interesting to note that, in the same study, it was shown that GFP expression was three times higher under the *c-fos* promoter than the CMV promoter. *In vivo* imaging of *c-fos* expression was tested with several imaging modalities (Wada et al., 2010). High expression in the hippocampus was demonstrated using chemical agents [the GABA antagonist pentylenetetrazol (PTZ) and kainic acid (KA)] in transgenic mice that had the reporter *c-fos*-LacZ fusion gene. The LacZ is a reporter gene that can be detected using histochemical staining in fixed brain slices. The *c-fos*-LacZ levels were detected as early as 30 min, peaked after 5 h, and returned to basal levels within 24 h (Smeyne et al., 1993). In another transgenic mice model, the LacZ gene was fused to a gene that encodes to the Tau protein in order to target the expressed protein to the neuronal processes. The fusion protein was under *c-fos* promoter regulation and was used to demonstrate functional neuronal circuits (Murphy et al., 2004).

Recently, a new approach for imaging changes in gene expression in real-time in deep brain areas associated with post-injury plasticity, in a living animal, was developed (Jouroukhin et al., 2014). A lentivirus that encodes to a yellow fluorescent gene (ZsYellow) driven by the *c-fos* promoter was introduced into rat cortical neurons. Using an advanced, fiber-based, optical imaging setup that enabled high-resolution imaging of deep brain areas, (Pelled et al., 2006) changes in gene expression were monitored in real-time (Figure 1). The results demonstrated that, in addition to functional MRI and electrophysiological changes that were characterized previously (Pelled et al., 2009; Li et al., 2011; Han et al., 2013), sensory deprivation induced changes in gene expression as well (Jouroukhin et al., 2014). This new approach can be expanded to a large array of promoters that can report with minimal invasiveness on changes in gene expression.

**Figure 1:**
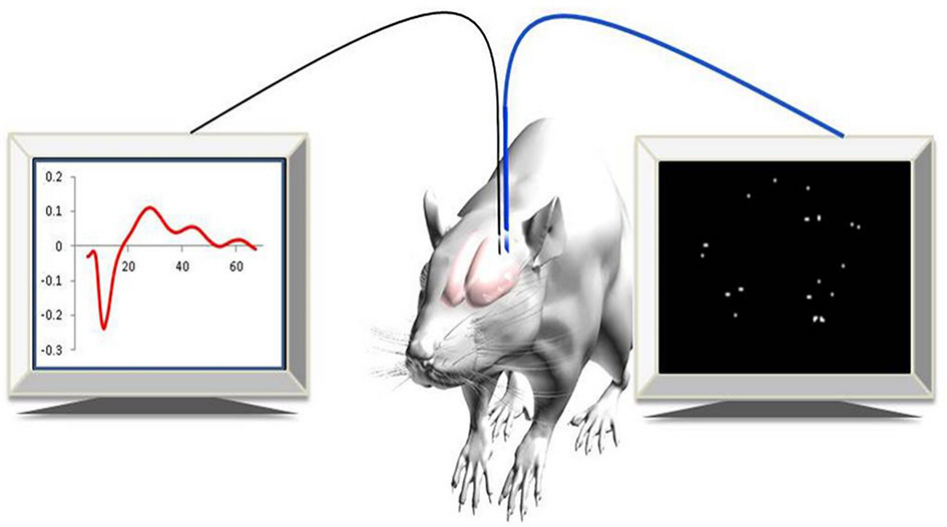
**Experimental paradigm**. Simultaneous optical and electrophysiology data were recorded in anesthetized rats expressing c-fos::ZsYellow1.

Alternatively, the optical reporter gene can be replaced with reporter genes that were developed for MRI. Rapid improvements in MRI hardware and techniques have led to increased spatial resolution (on the order of 50–100 μm for rodent imaging *in vivo*), which can be further enhanced by the expression of certain endogenous proteins that increase the MRI contrast (Duyn and Koretsky, 2011). Among a long list of MRI reporter genes that have been developed in the past two decades, there are reporters that are substrate-independent, such as ferritin(Genove et al., 2005; Cohen et al., 2007), the artificial gene called LRP (Lysine-Rich Protein) (Gilad et al., 2007a; Farrar et al., in press) and the human protamine-1 (hPRM-1) (Bar-Shir et al., 2014), which provide contrast based on Chemical Exchange Saturation Transfer (CEST) MRI (van Zijl and Yadav, 2011). These artificial reporters are evolving to become the most suitable for brain imaging, as their contrast is independent of whether the imaging substrate can cross the blood brain barrier.

## Conflict of interest statement

Assaf A. Gilad is a co-owner of SenCEST LLC. The authors declare that the research was conducted in the absence of any commercial or financial relationships that could be construed as a potential conflict of interest.
